# Proposing new body mass index and waist circumference cut-offs based on cardiometabolic risks for a Central Asia population: A feasibility study

**DOI:** 10.3389/fendo.2022.963352

**Published:** 2022-08-25

**Authors:** Aknur Kali, Arnur Gusmanov, Marat Aripov, Mei-Yen Chan

**Affiliations:** ^1^ Department of Medicine, School of Medicine, Nazarbayev University, Nur-sultan, Kazakhstan; ^2^ University Medical Center, School of Medicine, Nasarbayev University, Nur-sultan, Kazakhstan

**Keywords:** anthropometric measures, Body Mass Index, Waist Circumference, ethnicity, diabetes

## Abstract

**Background:**

Lower BMI cutoffs as compared to standard cut-offs have been recommended to reduce the risk of obesity-related co-morbidities in some ethnic populations (e.g. south Asian and Chinese populations). Recent attempts have also been made to establish ethnicity-specific BMI cutoffs to identify individuals affected with obesity in relation to type 2 diabetes risk in multi-ethnic populations based in the UK and North America. However, to date, there is yet to have any published work done to identify these cut-offs in Central Asia populations nor specify any difference for genders even though the fat distribution varies amongst the different ethnic groups as well as between the genders. To the best of the authors’ knowledge, this is the first study exploring new BMI and WC cut-offs in this population.

**Methods:**

To address this gap, we used a database of secondary care electronic health records from the National Research Cardiac Surgery Center to identify BMI and waist circumference cutoffs for obesity based on the risk of developing diabetes and other cardiometabolic disorders among 297 adults in Kazakhstan. Bivariate and multivariable logistic regression analysis were utilized to investigate the relationships between risk factors and type 2 diabetes mellitus (T2DM). BMI and WC thresholds were predicted using the Youden index.

**Results:**

For an equivalent age-adjusted and sex-adjusted incidence of type 2 diabetes at a BMI of 30·0 kg/m^2^ in White populations, we found higher BMI cutoffs for Kazakhstani women (30.5 kg/m^2^) but lower cut-offs for men (28·9 kg/m^2^). As for waist circumference, the cut-off points for females were 95cm and 104 cm for males.

**Conclusions:**

For Central Asia populations, the current recommended BMI and WC cutoffs may not be suitable and further work is needed to establish specific cut-offs for this population.

## Introduction

Rates of people with obesity has tripled over the last 30 years and the current pandemic has made it worse globally and in every country from developing to developed countries (1). The COVID-19 pandemic has forced many countries to impose circuit breakers, social distancing and reduced economic activity in various non-essential professions. These measures have inadvertently led to several disruptions in the food systems, such as changes in food intakes, physical activity patterns, as well as remote working environments, which could potentially exacerbate current trends in the prevalence of individuals with obesity (2).

Obesity puts many people at risk of developing metabolic syndrome, which further increases the risks of developing cardiovascular diseases complications, so it is important to identify obesity and manage or treat obesity (3). Extensive research has been conducted to determine appropriate BMI cut-off points for people affected with overweight and obesity based on ethnicity (4). A World Health Organization (WHO) expert consultation and National Institute for Health and Care Excellence (NICE) guidelines explained the debate over the interpretation of recommended body-mass index (BMI) cut-off points for determining overweight and obesity in Asian populations, as well as whether population-specific BMI cut-off points are required. They examined scientific evidence that suggests Asian populations have different relationships between BMI, body fat percentage, and health risks than Caucasian populations. The consultation indicated that the percentage of Asian people at high risk of type 2 diabetes (T2DM) and cardiovascular disease is significant at BMIs lower than the current WHO overweight cut-off point (25 kg/m^2^) (5, 6). According to studies conducted in Hong Kong, China, Japan, Thailand, and Singapore, the researchers recalculated BMI thresholds based on percentage body fat measurements, which are typically higher in Asians than in Caucasian populations, that is the Asians have higher percentage body fat at lower BMI cut-offs (7–11).

The WHO has determined two levels of central obesity in Europeans based on the risk of developing metabolic syndrome (12). A higher risk occurs at waist circumference (WC) cut-points of 94 cm for men and 80 cm for women, however, the risk is greatly increased at 88 cm in females and 102 cm in males. In Canada, Europe, and the United States higher cut-offs are used to define central obesity (12–14). For Asian populations, these cut offs for observed risk varies from 80 cm in women and 90 cm in men (10, 15, 16).

The term “Kazakhstanis” refers to the population of Kazakhstan. Kazakhstan, a geographically diverse and large country, gained its independence in 1991 after the disintegration of the Soviet Union. The population of Kazakhstan is multi-ethnic. From a total population of more than 19 million, ethnic Kazakhs constitute 69%, with 18.5% representing ethnic Russians, 3.3% ethnic Uzbeks, 1.5% ethnic Uigurs, while the remaining ethnic groups comprise 7.7% of the population (17).

In Central Asia including Kazakhstan, prevalence of overweight and obesity have almost doubled over the last 40 years (18). Furthermore, there is no available published work which has been done to determine these cut-offs in Central Asia populations or to clarify any differences in the sexes, even though fat distribution varies between ethnic groups as well as between genders. Therefore, our study aimed to explore the relationships amongst the waist circumference (WC) or body mass index (BMI) cut points in identifying the risks of cardiometabolic diseases in Kazakhstani adults.

## Materials and methods

### Study population and data collection

Anonymized patient records from National Research Cardiac Surgery center (NRCSC) in the Department of Interventional Cardiology were used in this study. These data and measurements are routinely collected as part of the inpatient treatment procedures. Data from 297 patients was utilized for analysis. Data for patients with congenital heart diseases was excluded. The study obtained ethics approval from Nazarbayev University Institutional Research Ethics Committee (NUSOM-IREC-NOV-2021-#25).

### Statistical analysis

All statistical analyses were performed using R software version 4.0.5. The prevalence and means of participants’ sociodemographic characteristics and obesity-related risk factors were calculated, and differences between groups were tested using the χ2-test for categorical variables and the Student’s t-test for continuous variables. Multivariable logistic regression was utilized to investigate the relationship between WC as an independent variable for type 2 diabetes mellitus (T2DM). Significance level for all statistical tests was set at 0.05. We used receiver-operator characteristics (ROC) curves to determine the optimal BMI or WC cut-points for identifying general or central obesity.

ROC curves were used to determine the optimal BMI and WC cut-offs based on the Youden index. A higher Youden index indicates greater accuracy. Since Youden’s statistic has the lowest mean squared error (MSE) and bias, it was more appropriate for use.

## Results

At baseline, secondary data from 297 patients were used for analysis. 65% of them were men and the mean age of all participants was 61 ± 9.8 years. The overall mean BMI, WC was 30.3 ± 5.85 kg/m^2^, 101.4 ± 13.65 cm, respectively. 33% of patients were diabetic (Type 2DM). Most patients (84.2%) had BMI more than 25 kg/m^2^ and 76.7% had a WC ≥94 cm in men and 90.4% women had WC ≥ 80 cm. 87% of patients had high blood pressure (>140 mmHg) while 81% of participants had ischemic heart disease. Majority of sample was identified as Kazakhs (61%), 34% as Russians and 5% as other race/ethnicity, which include Uzbeks, Tatars, and Koreans. Majority of the patients with diabetes had ischemic heart diseases and high blood pressure and most of them (33.6%) were males. 40.9% of participants with diabetes were aged over 60 years old. There was a significantly higher proportion of Russian patients with diabetes as compared to Kazakh patients (**Table 1**).

**Table 1:** Socio-demographic variables associated with type 2 diabetes mellitus (T2DM).

Variable	Diabetic patients (N=98)	Non-diabetic patients (N=199)	p-value
Age group, N* (%) <44 y.o 45-60 y.o >60 y.o	1 (5.6%) 32 (26.7%) 65 (40.9%)	17 (94.4%) 88 (73.3%) 94 (59.1%)	0.007
**Patient’s gender, N (%)** Male Female	65 (33.7%) 33 (31.7%)	128 (66.3%) 71 (68.3%)	0.83
BMI* categories, N (%) Normal (BMI<24.9 kg/m^2^) Overweight/Obese (BMI>25.0 kg/m^2^)	2 (4.3%) 96 (38.4%)	45 (95.7%) 154 (61.6%)	< 0.001
WC* (cm), mean ± SD	108.1 ± 12.1	98.1 ± 13.2	<0.001
**Ischemic heart disease, N (%)** **Yes** **No**	84 (35.4%) 13 (24.1%)	153 (64.6%) 41 (75.9%)	0.15
Systolic blood pressure**, N (%)** **High (≥140 mmHg)** **Normal (<140mmHg)**	88 (65.1%) 9 (23.1%)	164 (34.9%) 30 (76.9%)	0.201
**Smoking Status, N (%)** **Smokers** **Non-smokers**			0.89
Kazakh Russian Others	49 (27.2%) 41 (40.6%) 8 (50%)	131 (72.7%) 60 (59.4%) 8 (50%)	0.02
**Triglycerides (mmol/L), mean ± SD**	2.16 ± 1.29	1.53 ± 0.94	0.39
HDL* Cholesterol (mmol/L), mean ± SD	0.91 ± 0.82	1.01 ± 0.89	0.31
LDL* Cholesterol (mmol/L), mean ± SD	3.03 ± 0.98	3.27 ± 1.01	0.07
**Total Cholesterol (mmol/L) mean ± SD**	4.53 ± 1.18	4.71 ± 1.21	0.23

*BMI, body mass index; WC, waist circumference; HDL, high-density lipoproteins; LDL, low-density lipoproteins; SD, standard deviation. N denotes number of patients; % denotes percentage.

**Table 2** shows T2DM adjusted-model which included age, gender, ethnicity, and BMI. The adjusted odds of having diabetes were 8.6 times higher amongst those who were above 60 years old than those who were below 44 years old (adjOR = 8.6; 95% CI 1.44;16.82). Females had a slightly greater risk of developing T2DM (adjOR 1.09; 95% CI 0.61;1.96) than males but this did not reach statistical significance. Furthermore, those who were affected by increased body weight and/or obesity had a 14-fold increased risk as compared to those with normal BMI. However, the odds were reduced to about 2-fold when age and gender were adjusted (adjOR1.96; 95%CI 1.17; 4.34). Additionally, odds of having diabetes among other ethnic groups (adjOR 2.56; 95%CI 0.86; 7.87) was higher than Kazakhs.

**Table 2:** Multivariable logistic regression model for predicting type 2 diabetes mellitus using existing secondary data from NRCSC.

Variables	Unadjusted OR (95%CI)	p-value	Adjusted OR*(95%CI)	p-value
**Waist** **Circumference**	1.06 (1.03;1.09)	<0.001	1.05 (1.02; 1.08)	<0.001
**Age groups**				
<44 y.o	Ref		ref	
45-60 y.o	6.2 (1.12; 12.3)	0.08	3.93 (0.64; 7.72)	0.22
>60 y.o	11.7 (2.3; 21.4)	0.01	8.6 (1.44; 16.82)	0.05
**Gender**				
Male	Ref			
Female	0.92 (0.55;1.52)	0.73	1.09 (0.61; 1.96)	0.77
**Body mass index**				
Normal	Ref			
Overweight/obesity	14.02 (4.2; 8.75)	0.003	1.87 (0.97; 4.34)	0.01
**Ethnicity**				
Kazakh	Ref			
Russian	1.83 (1.09; 3.06)	0.02	1.21 (0.67; 2.16)	0.53
Others	2.67 (0.93; 7.64)	0.06	2.56 (0.86; 7.87)	0.088

*Adjusted for age, gender, BMI.

### WC and BMI cutoff points for abdominal and general obesity

**Figure 1:**
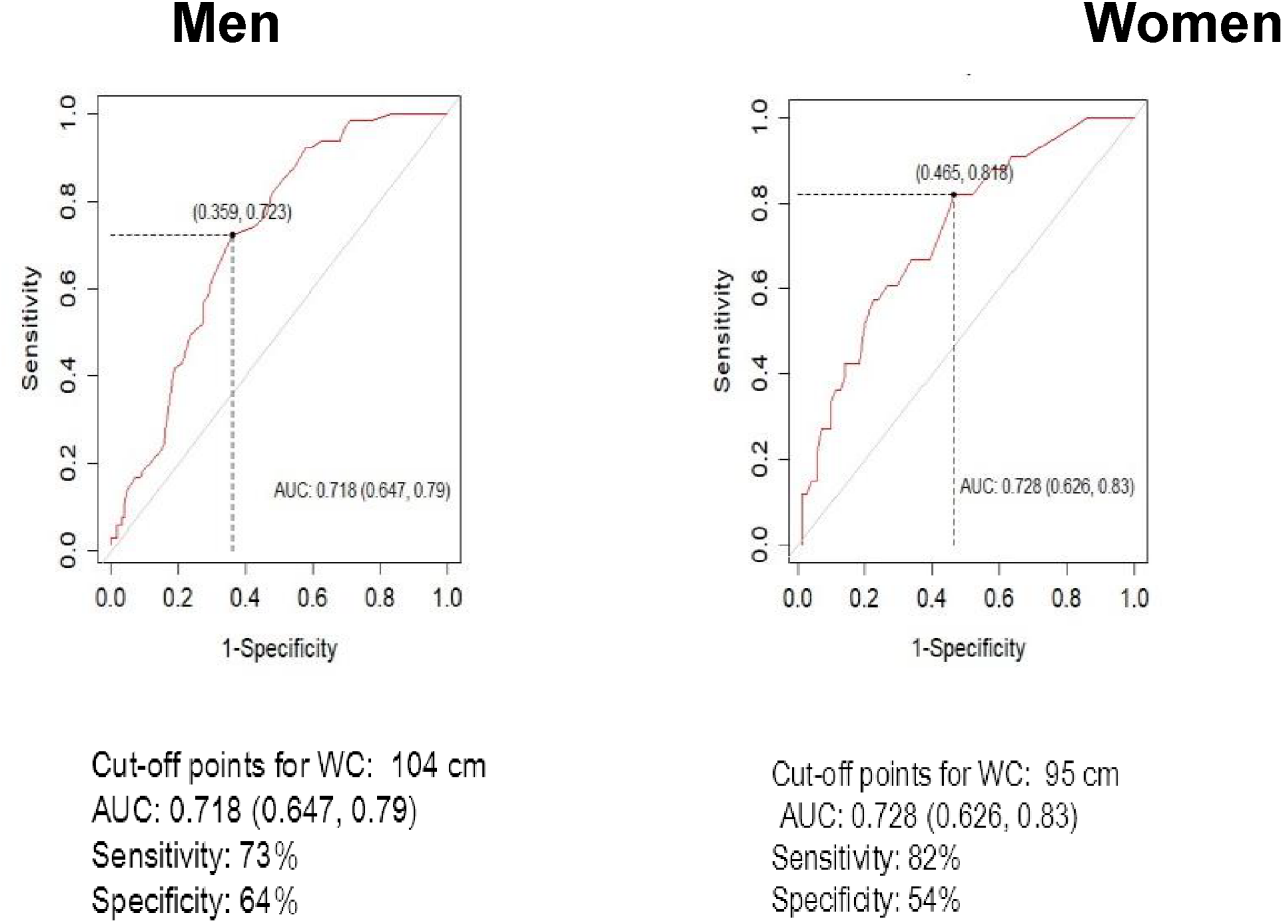
ROC analyses for waist circumference (WC) in the definition of Diabetes by sex.

**Figure 2:**
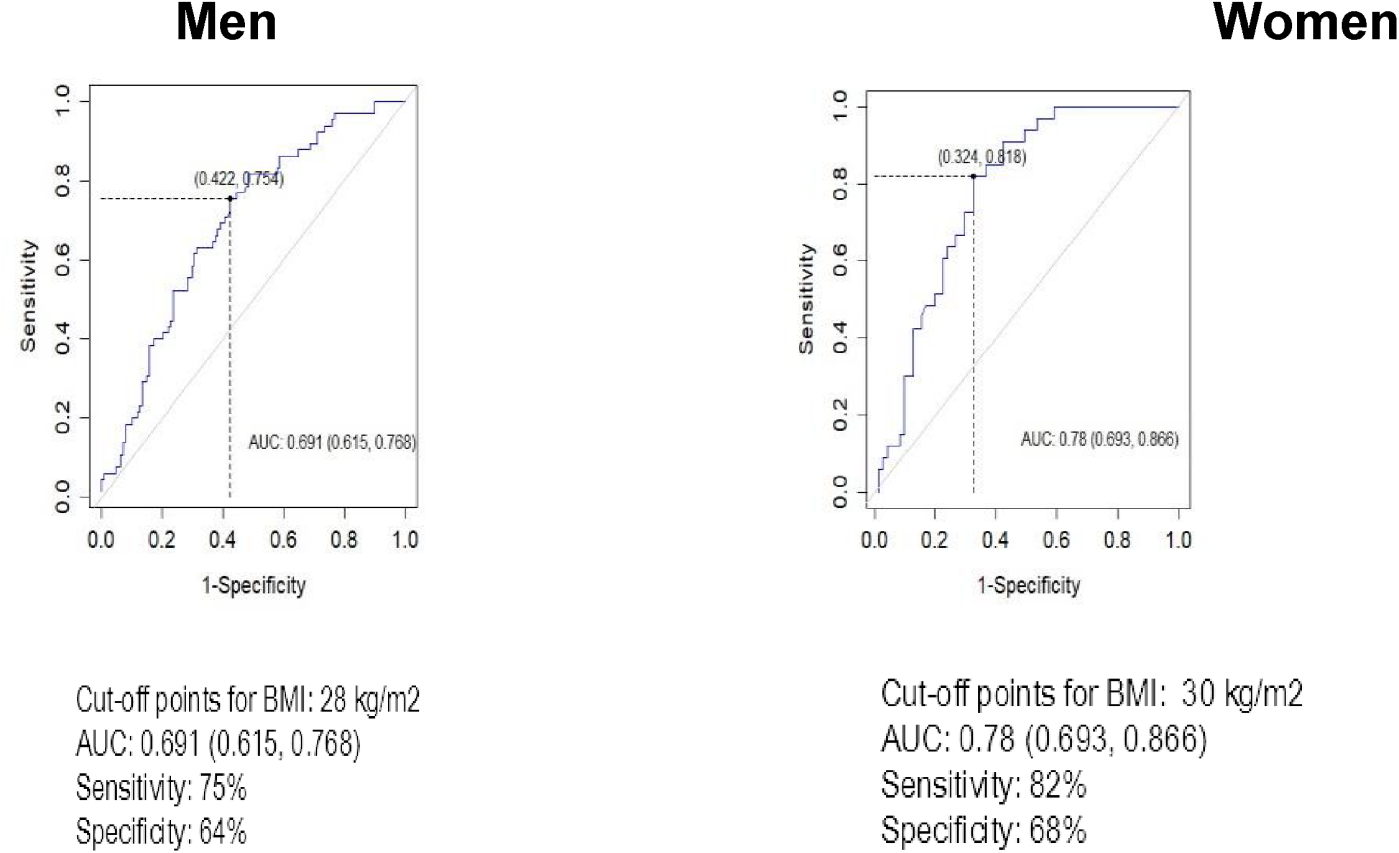
ROC analyses for body mass index (BMI) in the definition of Diabetes by sex.

Youden’s index was used to find the optimal cut-offs of BMI and WC in identification of diabetes. **Figure 1** depicts the ROC curves for WC as a screening indicator for diabetes separately for males and females. Higher WC cut-off was identified for males compared to females, 104 and 95 cm, respectively. WC cut-off for females demonstrated higher sensitivity (82% *vs* 73%), while specificity was higher among males (64% *vs* 54%). BMI cut-offs for diabetes were relatively close among sexes – 28 kg/m^2^ for males and 30 kg/m^2^ for females (**Figure 2**). While BMI cutoff for diabetes among females was more sensitive (82% *vs* 75%), specificity by gender deviated negligibly – 68% for women and 64% for men. The areas (95%CI) under the ROC curves for WC and BMI cut-offs for diabetes were 0.72 (0.65, 0.79) and 0.69 (0.62, 0.77) among males, while for females they accounted for 0.73 (0.63, 0.83) and 0.78 (0.69, 0.87), respectively.

**Figure 3:**
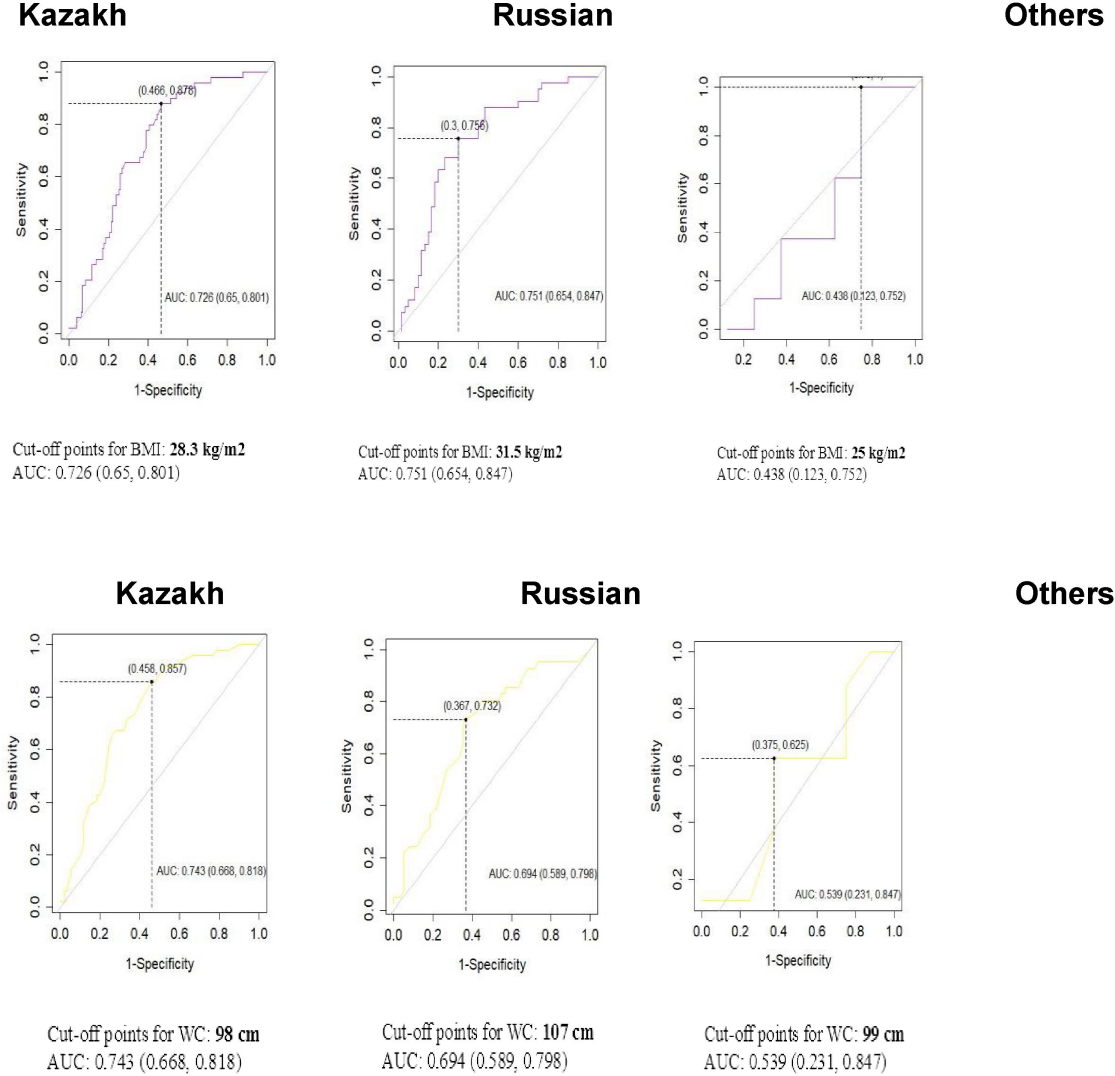
ROC analyses for body mass index (BMI) and waist circumference (WC) in the definition of Diabetes by ethnicity.

Estimated BMI and WC cutoffs to indicate the presence of diabetes were found to be higher for Russians compared to Kazakhs, 31.5 kg/m2 *vs* 28.3 kg/m2 for BMI cutoffs and 107 cm *vs* 98 cm for WC cutoffs, respectively (**Figure 3**). While for other ethnic groups the BMI cutoff accounted for the lowest value (25kg/m2) and WC cutoff was very close to that of ethnic Kazakhs (99 cm), the areas under the ROC curves for other ethnicities were nearby 50%.

## Discussion

An excessive storage of abdominal adipose tissue and obesity can be diagnosed by measuring WC and BMI, so certain cut-points are indicative of the diagnosis. The current available BMI and WC thresholds are measured mainly from Caucasian populations. Body proportions and fat distribution vary among ethnic groups so these cutoff points may not be applied to the other ethnic groups or populations (7). The IDF suggested thresholds utilized for the BMI and WC to determine general and abdominal obesity might be different among various ethnic groups (3, 7).

In our study, we investigated BMI and WC cut-points which can be used to determine cardiometabolic disease risk in Kazakhstani adults. Based on the observed findings of this study, the optimal BMI and WC cut-points for the Central Asian population are higher than for other Asian countries. Some WHO experts suggested that males’ thresholds in

Asian populations could be 90 cm and females’ thresholds could be 80 cm (10, 11). In Japan, various cut points have been proposed (8, 9), with the most recent studies recommending cutoffs of >85 to 90 cm for males and >80 cm for females. In China, optimal cut-points of >85 cm for males and >80 cm for females have been proposed (11). Similarly, for Asian countries, the optimal BMI thresholds for individuals who would like to lose excessive body weight or those being affected by obesity are lower (23-24.9 kg/m^2^) and (25 kg/m^2^), respectively (13, 14). Additionally, our study demonstrates differences in proposed BMI and WC cutoffs for ethnic Kazakhs and ethnic Russians. This finding underscores our hypothesis on the presence of different cutoffs among various ethnic groups and requires further investigation.

In this study, cut-off values for the anthropometric measurements were different in both sexes. Women were presented with higher BMI (30 kg/m^2^) cut points than men and the same results were obtained in another study which was conducted in Scotland where in females the mean BMI level was 2 kg/m^2^ higher than in males with T2DM (19). Additionally, in both gender the distribution of adipose tissue is different. In current study the greater level of abdominal fat was observed among women (Figure 5), however, research conducted by others have shown different results (20, 21). These studies revealed higher level of abdominal obesity and postulated that the higher abdominal adiposity in men might be related with the increased risk of cardiovascular diseases, glucose intolerance (21–26). The presence of a large proportion of women who are affected by central adiposity are probably attributed to the fact that in our sample, the female patients were with diagnosed with cardio-metabolic diseases and most of them were menopausal. In accordance with Mauvais-Jarvis (2018), menopausal are at greater risk of developing abdominal obesity and consequently developing cardiovascular disease (22).

The link between increased WC and cardio metabolic risk factors, which had been reported in developed countries, has rarely been considered in developing countries with different lifestyle of people, environments, and genetic characteristics and to the best of our knowledge, this is the first study to look at specific WC and BMI cut points in relation to waist circumference and cardiometabolic risk in a Central Asian population. Based on the area under the curve (AUC), the multivariate model in this study has good predictive power for using the cut-offs for type 2 Diabetes. The relationship between WC and risk factors such as age, sex, smoking habits, and serum lipids was explored in our model (27).

As expected, our data show that age was a statistically significant predictor of having T2DM (OR = 11.7, 95 percent CI: 2.3; 21.4, p=0.01). Previous research has found that elderly adults have more comorbid conditions and risk factors for T2DM than younger people (23). In our study smoking status was not a significant predictor for diabetes (p=0.89). Some evidence suggest that smoking expands the risk of having diabetes and it is associated with insulin resistance, dyslipidemia.

There were some limitations to this study as this was a feasibility study using a small sample. Firstly, our sample size was small (n=297) to perform further stratification analyses. Additionally, our study used data only from National Research Cardiac Surgery Center which was a hospital providing tertiary care, it is imperative to note that our findings should not be generalized to the entire Kazakhstani population. As a result, larger studies are required to back-up our findings and we are planning to do so in the future as part of this research. Considering the above-mentioned limitations, for future research, we will apply cluster sampling to allow us to explore some of the differences observed in rural versus urban populations as well as some of the minority ethnic groups.

## Conclusion and recommendations

The sex-disaggregated waist circumference thresholds for males and females were 10 cm and 15 cm and the BMI cut-offs were 3 kg/m^2^ and 5 kg/m^2^ higher than those currently recommended by WHO. In this feasibility study, both ethnic Kazakhs and Russians were found to have higher BMI cut-offs in comparison with current cut-off values suggested by WHO for Asian population – 25 kg/m^2.^ Additionally, differences in BMI and WC cut-offs between ethnic Kazakhs and Russians require further investigations. Therefore, our findings suggest that further larger studies should be conducted to define optimal BMI and WC cut-points for entire Kazakhstani population.

## Data availability statement

The raw data supporting the conclusions of this article will be made available by the authors, without undue reservation.

## Ethics statement

This study was reviewed and approved by Nazarbayev University Institutional Research Ethics Committee (NUSOM-IREC-NOV-2021-#25). The patients/participants provided their written informed consent to participate in this study.

## Author contributions

Conceived and designed the study: MYC. Performed the study: AK, MA. Analyzed the data: AK, AG, MYC. Interpreted the results: AK, AG, MA and MYC. Wrote the paper: AK, AG, MYC. Principal Investigator of this study and supervised this study: MYC. All authors contributed to the article and approved the submitted version.

## Conflict of interest

The authors declare that the research was conducted in the absence of any commercial or financial relationships that could be construed as a potential conflict of interest.

## Publisher’s note

All claims expressed in this article are solely those of the authors and do not necessarily represent those of their affiliated organizations, or those of the publisher, the editors and the reviewers. Any product that may be evaluated in this article, or claim that may be made by its manufacturer, is not guaranteed or endorsed by the publisher.
